# Improving communication skills in the Southeast Asian health care context

**DOI:** 10.1007/s40037-014-0121-4

**Published:** 2014-05-28

**Authors:** Mora Claramita, Astrid Pratidina Susilo

**Affiliations:** 1 Department of Medical Education Faculty of Medicine, Gadjah Mada University, Radiopoetro Building 6th Floor, Jalan Farmako Sekip Utara, Yogyakarta, 55281 Indonesia; 2 Faculty of Medicine, Mulawarman University, Jl. Kerayan, Kampus Gn. Kelua, Samarinda, 75119 East Kalimantan Indonesia

**Keywords:** Communication skills training, Intercultural communication, Interprofessional communication, Informed consent, Informed and shared decision making

## Abstract

The aim of these two PhD thesis are to develop a guideline on doctor-patient communication skills based on cultural characteristics of Southeast Asian context and to develop communication skills training for nurses to enhance their contribution to the informed consent and shared decision making process, in the same context. These studies started with qualitative methods; including grounded theory methodology, by exploring doctors’, patients’, medical students’ and nurses’ perceptions on the current and desired communication skills in which influenced by culture. Based on the results, we design communication skills training and evaluate the training with quantitative methods, using pre and post test studies. Southeast Asian desired ideal partnership style in communicating with their doctors. More emphasize on basic skills such as listening to subtle non-verbal cues are needed for doctors and nurses. A guideline on doctor-patient communication tailored to local culture was developed as well as training for nurses using 4CID design to enhance their contribution to the shared decision making process. To promote two-way interaction between doctors and patients and between health professionals require mastering basic skills in communicating with people, such as explorations on the unspoken concern. In a culturally hierarchical context of Indonesia, this two-way interaction is quite a challenge. To generalize our studies to other culture, more studies with rigorous methods should follow. To promote the use of basic skills in communicating with patients to approach the desired partnership communication style in Southeast Asian context, we need to use local evidences.

## Introduction

Communicating with patients is considered to be central to the clinical abilities of health professionals world-wide and its correct application is essential in different phases of clinical care, including during the decision-making process [1]. Studies on communication in health care have been mostly conducted in the Western world. They might limit the validity of the evidence for the Southeast Asian context which is characterized by both a strong hierarchical and communal culture [2]. In a strong hierarchical culture, the power distance among people is large, including that between doctors and patients and doctors and nurses [3]. In a communal culture, there is a strong involvement of community and family in individual decision-making in health care. Additionally, in most countries in Southeast Asia, health care systems are overburdened and the education system for health care professionals suffers from lack of standardization [4]. Lack of time for consultation is the number one barrier in communicating properly with patients because of the high patient load due to a system which is not ‘appointment-based’.

In this PhD report we combine the findings of two dissertations that aimed to explore how the characteristics of the Southeast Asian culture and its health care system influence the clinical decision-making process and also more specifically with regard to the informed-consent process. We used these insights to design and evaluate improvement strategies. The first dissertation aimed to develop a doctor-patient communication guideline based on Southeast Asian cultural characteristics [5] while the second one aimed to develop a communication skills training for nurses to enhance their contribution during the informed consent process [6].

## Methods

Each dissertation consists of a series of qualitative and quantitative studies using different approaches such as grounded theory and case study, as well as various methods such as survey and pre-post test design. Interviews, focus group discussions, and observations were conducted to explore perceptions of patients, family members, doctors, medical students, nurses, and hospital managers regarding (1) doctor-patient communications, (2) informed consent as a reflection of the decision-making process, and (3) the role of nurses within this decision-making process.

Based on the results, we developed approaches for improvement which are tailored to the needs of Southeast Asia. The first dissertation resulted in a doctor-patient communication guideline, and the introduction of a guideline using a participatory approach for medical teachers was evaluated. The second dissertation developed a communication skills course to prepare nurses to be patient advocates. The course was piloted in an inter-professional setting and evaluated using a questionnaire, pre-post test, and focus group discussions.

## Results

Patients felt that medical residents’ ability to communicate is worse than their expectations. However, medical residents who were taught communication skills using Western guidelines in a problem-based learning (PBL) format during their undergraduate medical curriculum perceived themselves to have the same low scores as the patients’ perceptions. Whereas the medical residents without undergraduate training in communication skills in a non-PBL curriculum perceived themselves to have higher scores than patients’ perceptions. In this regards, there is an urgent need for better and more systematic communication skills training, especially training accommodating local cultural characteristics [7].

Both doctors and patients indicated they would prefer a more partnership-oriented style of communication; however, the commonly practised style was one-way [8]. A one-way communication style was practised because of a lack of time for consultation related to the poorly structured health care system, patients were not prepared for a dialogical communication because of the culturally hierarchical gap between doctors and patients and also because of doctors’ lack of training in communication skills [9]. To apply the desired partnership style of communication with patients, our findings suggest that doctors need to use more the core communication skills, which turned out to be the key to addressing cultural aspects of Southeast Asian people: (1) When doctors greet their patients they should explore what the patients would like to be called. In Indonesia, the ‘term of address’ may be as close as if they were greeting a member of their own family to provide a warm rapport and to avoid the cultural hierarchical gap; (2) Paying attention to any subtle, Southeast Asian nonverbal cues is important. What the patient says may not represent what they are thinking, because naturally they would maintain harmony by being polite; (3) The strong influence of family members in clinical decision-making within a communal culture requires the doctor to balance the patient’s and family’s opinions, without neglecting patient preferences; (4) Almost everyone uses alternative medicine, so this should be taken into consideration when discussing the care plan [10, 11]. A guideline for communicating with Southeast Asian patients accommodating their cultural characters is called: The Greet-Invite-Discuss guideline [12].

During the informed consent process, patient autonomy is frequently challenged by the cultural context. Patients are not able to address their concerns to the doctors who are considered to be higher in hierarchy; additionally the strong involvement of family often overrides the patient’s autonomy, rendering it difficult for patients to make independent choices [6]. On the other hand, doctors often use informed consent to protect themselves from a potential legal suit [13]. Although nurses can act as patient advocates, their role during the informed consent process is also influenced by the hierarchical relationship with the doctors and time constraints in the workplace. Nurses are frequently overburdened with tasks delegated by doctors [14]. Doctors and nurses have similar views on the barriers during the informed consent process but they have different perceptions about the potential of the nurse’s role within the process [15].

To be able to act as advocates in such a challenging context, health professionals should integrate clinical, legal and ethical knowledge with the communication skills [14]. A course to help professionals learn to be patient advocates was piloted for inter-professional participants such as nurses and doctors [16]. Leary’s Rose, a model to map different hierarchical positions in a negotiation process, was used as an educational tool to help participants face hierarchical encounters [17, 18]. The Four-Components Instructional Design model (4C/ID), a model that emphasizes the importance of using whole and authentic learning tasks, was used to design the course [16, 19]. Both models are based on Western educational principles, but their use was tailored to the local situation. Course evaluations showed retention of knowledge and transfer to practice [20].

## Discussion

Patients want their communication with health professionals to be as ideal as possible, characterized by trust, equal participation and two-way dialogue during shared clinical decision-making. In our studies, performed in a far Southeast Asian society like Indonesia, patients demonstrated similar desires to those of patients in Western societies [5]. In the culturally hierarchical context of Southeast Asia, understanding these cultural characteristics is essential to communicate effectively with patients and hopefully improve health outcomes. More emphasis on the core of communication skills is needed and training them is essential in improving doctor-patient communication skills. Additionally, training nurses as patient advocates will improve patient autonomy as nurses can help to address patients’ concerns in the decision-making process [6].

Although the research for both our dissertations was situated in an Indonesian setting (Central Java, East Java and East Kalimantan) we feel that our findings can be transferable to other cultures which are characterized by strong hierarchical and communal cultures.

Both dissertations focused on the exploration of the current conditions. The recommendations formulated by our dissertations should be tested with a bigger population and perhaps in a more experimental fashion, for instance using a randomized trial in other institutions and multi-professionals contexts and employing direct observation in the assessment process.

## Conclusion

A partnership approach to doctor-patient communication in a Southeast Asian context can be achieved by a stronger emphasis on the core of communication skills. Inter-professional collaboration is helpful to ensure that patient autonomy is respected during the informed consent process. Tailoring evidence from the Western world to a local cultural context has proven to be an effective approach to designing culturally sensitive educational programmes aimed at training communication skills for health professionals in Southeast Asian settings.

## Advice for PhD students

A PhD process is a journey, starting with opening the mind to new information. This can only be done by listening to and observing every tiny piece of evidence which may come from any source and may include both conducting a thorough literature review and listening to unspoken facts. Careful deep and wide reflection should be the basis for action targeted on a better life for people. Communicating all of this is another journey which promises endless learning for mankind.
